# Analysis of risk factors for acute pancreatitis complicated with pancreatic sinistral portal hypertension and construction of predictive model

**DOI:** 10.3389/fphys.2023.1256615

**Published:** 2024-01-08

**Authors:** Xin Zhao, Tian-Yang Mao, Kang-Yi Jiang, Qing-Yun Xie, Jie Yang, Bo Du, Zhi-Xu Wang, Jin-Qiang Fu, Feng-Wei Gao, Ze-Hua Lei

**Affiliations:** ^1^ Department of Hepatopancreatobiliary Surgery, The People’s Hospital of Leshan, Leshan, Sichuan, China; ^2^ Diagnosis and Treatment Center for Liver, Gallbladder, Pancreas and Spleen System Diseases of Leshan, Leshan, Sichuan, China; ^3^ Liver Transplantation Center, State Key Laboratory of Biotherapy and Cancer Center, West China Hospital, Sichuan University and Collaborative Innovation Center of Biotherapy, Chengdu, Sichuan, China

**Keywords:** acute pancreatitis, pancreatic sinistral, portal hypertension, nomogram, predictive model

## Abstract

**Objective:** Pancreatic sinistral portal hypertension (PSPH) is a common complication of acute pancreatitis (AP) and can cause massive gastrointestinal bleeding, which is one of the causes of AP-related mortality. However, there is currently no predictive model for AP concurrent with PSPH. This study aimed to identify the risk factors for AP concurrent with PSPH and use these factors to build a related predictive model.

**Materials and methods:** We collected clinical data from 282 patients with AP. 192 patients were used as a training group and 90 patients as a validation group. Univariate and multivariate analyses were used to identify independent risk factors for AP complicated with PSPH, and then a nomogram was established. The models are cross verification and Internal verification. The predictive ability and accuracy of the model were evaluated based on the working curve of the subjects and the calibration curve, respectively. The clinical value of the model was evaluated using decision curve analysis (DCA).

**Results:** The univariate analysis revealed significant differences in the occurrence of PSPH with respect to sex, recurrent AP, history of hypertension, smoking history, patency of the splenic vein, pancreatic necrosis or pancreatic pseudocyst formation, the most significant site of pancreatic swelling, presence of a Dmure D polymer, MCTSI, and involvement of lipase and amylase. The logistic multivariate regression analysis showed that male sex, splenic-vein stenosis or occlusion and swelling were located in the body-tail, and MCTSI was an independent risk factor for PSPH. The nomogram and ROC curve were constructed. The area under the working curve of the subjects was 0.91, and the sensitivity and specificity were 82.5% and 89.1%, respectively. In the validation group, the C-index is 0.826. The nomogram was internally validated using 1,000 bootstrap samples, and the c-index was 0.898. The calibration curve demonstrated that the predicted probability was concordant with the observed probability, and the DCA confirmed that the model had robust clinical utility.

**Conclusion:** Male sex, splenic-vein stenosis or occlusion, recurrent AP, and swelling are located in the body-tail, and MCTSI is an independent risk factor for the occurrence of PSPH. The predictive model developed for AP complicated with PSPH may serve toward developing preventive and therapeutic approaches for PSPH.

## Introduction

Acute pancreatitis (AP) is a local inflammatory disease of the exocrine glands of the pancreas and is one of the most common digestive-tract diseases. The global incidence rate is (4.9–73.4)/100000 (Boxhoorn et al., 2020; Spagnolo et al., 2022). For AP patients with systemic inflammatory-response syndrome and/or multiple-organ failure, the disease progresses rapidly, the treatment is difficult, and the case fatality rate is as high as 20%–40% (Lee and Papachristou, 2019; Trikudanathan et al., 2019). Peripancreatic fluid accumulation, acute necrotic accumulation, pancreatic pseudocyst formation, pancreatic sinistral portal hypertension (PSPH), gastrointestinal or abdominal bleeding, and intestinal fistula are common complications of AP (Jiang et al., 2020; Vogel et al., 2022).

PSPH is a common complication of AP and accounts for 5%–10% of cases of extrahepatic portal hypertension (Gyoten et al., 2017; Ru et al., 2020). It results from pancreatic diseases that affect the portal vein and the associated branches. The most commonly affected vein in pancreatic diseases leading to PSPH is the splenic vein, which may experience vascular obstruction and blood-reflux disturbance. This vascular pathology can lead to splenomegaly and increased splenogastric venous pressure, which are the main clinical manifestations of regional portal hypertension. PSPH is a common complication of AP and the splenomegaly accompanied by hypersplenism and massive gastrointestinal hemorrhage caused by PSPH is one of the causes of death in AP patients. At present, about 4%–17% of patients with PSPH will develop gastrointestinal bleeding, and about 1.2%–14.5% will die as a result (Ru et al., 2020).

Therefore, early identification and intervention, or effective treatment of AP complicated with PSPH is of great significance to improve the prognosis and minimize associated medical costs. Although the risk factors for AP complicated with PSPH have recently been probed by several groups, no optimal predictive model for this condition has been established (Wang et al., 2022; Yu et al., 2022). Thus, this study aimed to identify the major risk factors and use them to construct a predictive model of AP complicated with PSPH.

## Materials and methods

### Patients selection

**FIGURE 1:**
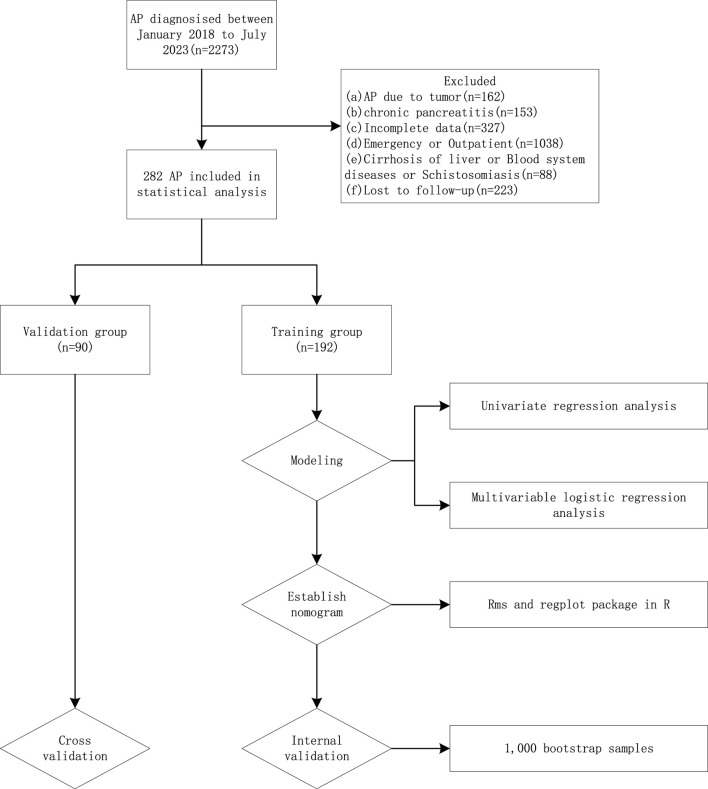
Study design flowchart of specific patient screening process.

Clinical data from 2,572 patients with AP registered at the Leshan People’s Hospital between January 2018 and July 2023 were collected retrospectively. The inclusion criteria were as follows: 1) a clear history of non-tumor–related AP; 2) no history of cirrhosis, hematological diseases, or schistosomiasis; 3) cases with complete clinical data; 4) inpatients; and 5) cases that were followed up. The exclusion criteria were 1) history of pancreatitis caused by tumor; 2) presence of chronic pancreatitis; 3) history of cirrhosis, hematological diseases, or schistosomiasis; 4) incomplete clinical data; 5) emergency or outpatient cases; and 6) cases that were lost during the follow-up (Figure 1). Finally, 292 patients were included, of which 192 patients from January 2018 to December 2022 were used as the training group to build the model, and 90 patients from January 2023 to July 2023 were used as the validation group to verify the model (Table 1).

**TABLE 1:** Demographic characteristics of the study population.

Parameters	Patients (*n* = 192)
Sex	
Male	112 (58.3)
Female	80 (41.7)
Age (y)	51.73 ± 14.56
BMI	
Not overweight	149 (77.6)
Overweight	43 (22.4)
Recurrent AP	
Yes	71 (37)
No	121 (63)
Etiology	
Alcoholic	14 (7.3)
Biliary	72 (37.5)
HTG	57 (29.7)
Other unknown	49 (25.5)
Hypertension	
Yes	41 (21.4)
No	151 (78.6)
Diabetes	
Yes	45 (23.4)
No	147 (76.6)
Smoking	
Yes	87 (45.3)
No	105 (54.7)
Drinking	
Yes	77 (40.3)
No	115 (59.9)
Splenic-vein	
Stenosis or Occlusion	37 (19.3)
Normal	155 (80.7)
PNC	
Yes	96 (50)
WON	84 (43.8)
WON and infection	3 (1.5)
PPs	9 (4.7)
No	96 (50)
SSPS	
Head	86 (44.8)
Body-tail	106 (55.2)
TT (seconds)	17.34 ± 1.70
MCTSI	6 (6,8)
Fibrinogen (g/L)	4.79 (3.63, 5.73)
D-dimer (mg/L FEU)	1.72 (0.68, 1.82)
INR	1.03 (0.96, 1.1)
Triglyceride (mmol/L)	4.63 (1.54, 7.07)
Total cholesterol (mmol/L)	5.84 (4.17, 6.04)
PLT (×10^9^/L)	168.5 (132.25, 228.25)
CRP (ng/mL)	67.58 (12.88, 153.23)
PCT (ng/mL)	0.76 (0.18, 2.08)
Serum lipase (u/L)	813.52 (210.75, 894.52)
Serum amylase (u/L)	554.5 (136.00, 796.25)

BMI, Body mass index; AP, Acute pancreatitis; HTG, hypertriglyceridemia; PNC, Pancreatic necrosis or cyst; WON, walled-off necrosis; PPs, pancreatic pseudocyst; SSPS, Significant site of pancreatic swelling; TT, Thrombin time; MCTSI, The modified CT severity index; INR, International standardized ratio; PLT, Platelet; CRP, C-reactive protein; PCT, Procalcitonin.

### Diagnosis and definitions

The diagnosis of AP was based on persistent epigastric pain, serum amylase level > 3-folds of the normal level, and computed tomography (CT) or magnetic resonance imaging (MRI) findings suggestive of AP (Banks et al., 2013). The diagnosis of PSPH was based on enhanced CT or MRI findings indicating portal-vein obstruction, solitary gastroesophageal varices, splenomegaly, and the absence of cirrhosis or abnormal liver function. Splenic-vein occlusion was defined as complete occlusion with no blood flow, whereas the thinnest point of the splenic vein is defined as stenosis if it is less than or equal to 50% of the normal mean value (Easler et al., 2014; Yu et al., 2022). Body mass index (BMI) ≥ 25 kg/m^2^ was defined as overweight, and BMI <25 kg/m^2^ was defined as not overweight (Caballero, 2019). Patients with a prior history of acute pancreatitis admitted to the hospital for treatment were defined as Recurrent AP (Hu et al., 2022).

### Clinical-data collection

#### General information

The information about patient sex, age, BMI, and any history of pancreatitis, etiology of pancreatitis, hypertension, diabetes, smoking, or drinking was collected.

#### CT or MRI findings

On admission or diagnosis with pancreatitis, all the patients underwent CT or MRI to assess the patency of the splenic vein. The imaging was used to detect any thrombus, stenosis, or occlusion, as well as necrotic accumulation or pseudocyst formation, and identify the most significant site of pancreatic swelling. The severity of pancreatitis was graded by radiographers according to the modified CT severity index (MCTSI).

### Serological tests

Serological tests were performed on admission, and thrombin time (TT), international standardized ratio (INR), and D-dimer, fibrinogen, triglyceride, total cholesterol, platelet (PLT), C-reactive protein (CRP), procalcitonin (PCT), blood lipase, and hemodiastase levels were recorded.

### Follow-up

For patients with unclear history of pancreatitis, cirrhosis, hematological diseases, and schistosomiasis, data were obtained by telephone follow-up. Follow up with CT or MRI every 3–6 months after discharge, with a duration of 12 months.

### Statistical analysis

The SPSS (v26.0) software was used for statistical analysis. For normally distributed measurement data, descriptive statistics were presented as mean ± SD, and group comparisons were made using the t-test. For skewed distribution data, descriptive statistics were presented as median (±interquartile range), and group comparisons were made using the Mann-Whitney U test. Categorical data were presented as number of cases (percentage), and group comparisons were made using the chi-squared test. The factors found to be related to the occurrence of PSPH in the univariate analysis were included in the multivariate logistic regression model analysis to identify the independent risk factors for PSPH. The identified independent risk factors were used to construct an nomogram and calibration curve by using “rms,” and “regplot” programs in the R (v4.2.2) software, respectively. “pROC,” “plotROC,” and “rmda” were used to draw the receiver operating characteristic (ROC) and decision curves of the predictive model, and the area under the ROC curve (AUC) was calculated to evaluate the effectiveness of the predictive model. Finally, 1,000 bootstrap samples were used for internal verification.

## Results

In the training group, the patients were divided into PSPH (*n* = 63) and non-PSPH (*n* = 129) groups according to the occurrence of PSPH. Univariate analysis showed that age, BMI, etiology of pancreatitis, history of diabetes, history of drinking, TT, INR, platelet count, or serum level of fibrinogen, CRP, triglyceride, or total cholesterol had no significant effect on the occurrence of PSPH (*p* > 0.05). Sex (*p* = 0.010), hypertension history (*p* = 0.041), recurrent AP (*p* < 0.001), smoking history (*p* = 0.021), splenic-vein stenosis or occlusion (*p* < 0.001), pancreatic necrosis or cyst (*p* < 0.001), significant site of pancreatic swelling (*p* = 0.001), D-dimer (*p* = 0.014), MCTSI (*p* < 0.001), serum lipase (*p* = 0.015), and serum amylase (*p* = 0.005) had significant effect on the occurrence of PSPH (*p* < 0.05) (Table 2).

**TABLE 2:** Comparisons between PSPH and control groups.

Characteristics	PSPH group (*n* = 63, %)	Control group (*n* = 129, %)	X^2^/t/z	*p* value
Sex			6.616	0.010
Male	45 (71.4)	67 (51.9)		
Female	18 (28.6)	62 (48.1)		
Age (yr)	50.00 ± 14.33	52.58 ± 14.65	1.155	0.250
BMI			0.167	0.683
not overweight	50 (79.4)	99 (76.7)		
overweight	13 (20.6)	30 (23.3)		
Recurrent AP			16.359	*p* < 0.001
Yes	36 (57.1)	35 (27.1)		
No	27 (51.9)	94 (72.9)		
Etiology				
Alcoholic	7 (5.4)	7 (11.1)	2.024	0.155
Biliary	48 (37.2)	24 (38.1)	0.014	0.905
HTG	44 (34.1)	13 (20.6)	3.681	0.055
Other unknown	30 (23.3)	19 (30.2)	1.061	0.303
Hypertension			4.183	0.041
Yes	8 (12.7)	33 (25.6)		
No	55 (87.3)	96 (74.4)		
Diabetes			0.007	0.932
Yes	15 (23.8)	30 (23.3)		
No	48 (76.2)	99 (76.7)		
Smoking			5.296	0.021
Yes	36 (57.1)	51 (39.5)		
No	27 (42.9)	78 (60.5)		
Drinking			3.234	0.072
Yes	31 (49.2)	46 (35.7)		
No	32 (50.8)	83 (64.3)		
Splenic-vein			66.076	*p* < 0.001
Stenosis or Occlusion	33 (52.4)	4 (3.1)		
Normal	30 (47.6)	125 (96.9)		
PNC			35.934	*p* < 0.001
Yes	51 (81.0)	45 (34.9)		
No	12 (19.0)	84 (65.1)		
SSPS			12.024	0.001
Head	17 (27.0)	69 (53.5)		
Body-tail	46 (73.0)	60 (46.5)		
TT (seconds)	15.33 ± 1.64	17.34 ± 1.74	0.048	0.961
MCTSI	8 (8,9)	6 (6,6)	7.520	*p* < 0.001
Fibrinogen (g/L)	4.86 (3.77, 5.40)	4.73 (3.55, 5.74)	0.446	0.656
D-dimer (mg/L FEU)	1.82 (1.13, 1.82)	1.63 (0.63, 1.82)	2.446	0.014
INR	1.06 (0.99, 1.11)	1.01 (0.96, 1.10)	1.638	0.101
Triglyceride (mmol/L)	6.08 (1.70, 6.62)	3.72 (1.53, 7.07)	0.418	0.676
Total cholesterol (mmol/L)	5.84 (4.09, 5.95)	5.84 (4.41, 6.54)	0.839	0.401
PLT (×109/L)	162.00 (120.50, 222.50)	173.00 (138.00, 232.00)	0.671	0.502
CRP (ng/mL)	84.76 (21.17, 157.70)	61.83 (9.22, 141.04)	1.772	0.076
PCT (ng/mL)	1.61 (0.43, 2.09)	0.53 (0.17, 2.08)	1.772	0.076
Serum lipase (u/L)	813.52 (143.00, 813.52)	813.52 (226.00, 1030.00)	2.423	0.015
Serum amylase (u/L)	287.00 (101.00, 727.59)	625.49 (163.00, 1208.00)	2.807	0.005

PSPH, pancreatic sinistral portal hypertension; BMI, body mass index; AP, acute pancreatitis; HTG, hypertriglyceridemia; PNC, pancreatic necrosis or cyst; SSPS, significant site of pancreatic swelling; TT, thrombin time; MCTSI, The modified CT, severity index; INR, international standardized ratio; PLT, platelet; CRP, C-reactive protein; PCT, procalcitonin.

The above indices were used to perform a logistic multivariate regression analysis, which revealed male sex (*p* = 0.010), recurrent AP (*p* = 0.006), splenic-vein stenosis or occlusion (*p* < 0.001), swelling are located in the body-tail (*p* < 0.001), and MCTSI (*p* < 0.001) as independent risk factors for PSPH (Table 3). The above indexes were included in the verification group and analyzed with the training group, and the results were not statistically significant (*p* > 0.05) (Table 4).

**TABLE 3:** Logistic multivariate regression analysis of the candidate indicators for PSPH.

Parameters	OR (95% CI)	*p* value
Male Sex	0.184 (0.051–0.667)	0.010
Recurrent AP	4.673 (1.554–14.057)	0.006
Splenic vein narrowing or blockage	12.7 (3.354,48.09)	*p* < 0.001
SSPS	7.662 (2.56–22.93)	*p* < 0.001
MCTSI	1.862 (1.319–2.63)	*p* < 0.001

AP, acute pancreatitis; SSPS, significant site of pancreatic swelling; MCTSI, The modified CT, severity index; SSPS, significant site of pancreatic swelling.

**TABLE 4:** Comparisons between Training group and Validation groups.

Parameters	Training group (%)	Validation group (%)	*p* value
Sex			0.255
Male	112 (58.3)	46 (51.1)	
Female	80 (41.7)	44 (48.9)	
Recurrent AP			0.68
Yes	71 (37)	31 (34.4)	
No	121 (63)	59 (65.6)	
Splenic-vein			0.319
Stenosis or Occlusion	37 (19.3)	22 (24.4)	
Normal	155 (80.7)	68 (75.6)	
SSPS			0.768
Head	86 (44.8)	42 (46.7)	
Body-tail	106 (55.2)	48 (53.3)	
MCTSI	6 (6, 8)	6 (5.5, 8)	0.475

## Construction of a nomogram

**FIGURE 2:**
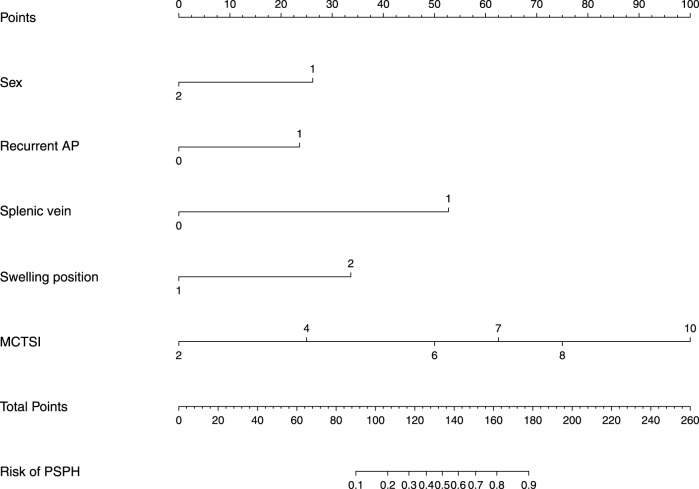
Nomogram for predicting incidence rate among patients with AP. AP, Acute pancreatitis; MCTSI, The modified CT severity index.

Based on the results of the logistic multivariate regression analysis, a nomogram that comprised five important factors for predicting the occurrence of PSPH (sex, recurrent AP, patency of the splenic vein, site of swelling, and MCTSI) was constructed. Each value of these variables was assigned a score on the scale axis, and the total score was calculated by summing up each score. By projecting the total score on the probability axis, the likelihood of PSPH occurrence was estimated (Figure 2).

**FIGURE 3:**
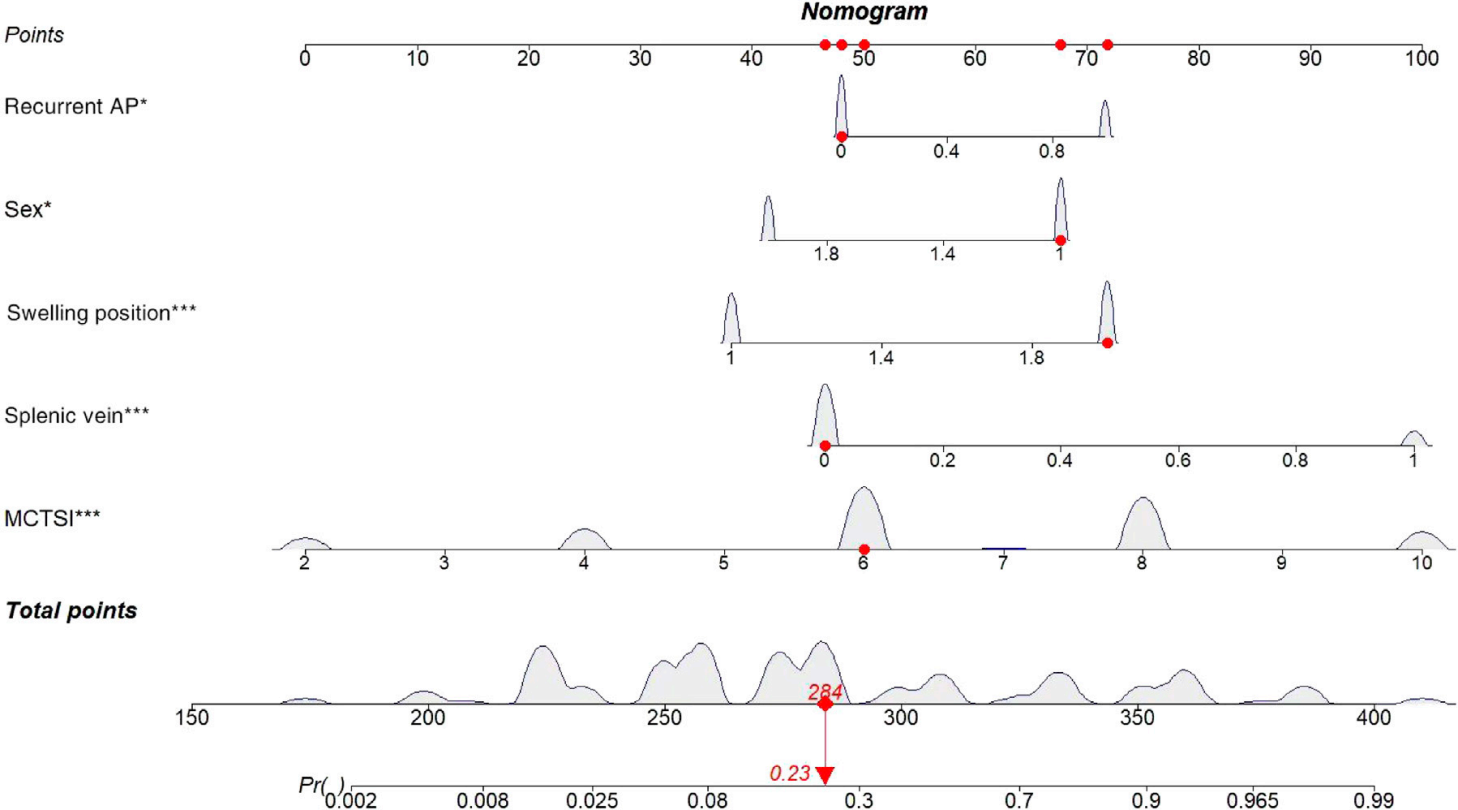
Dynamic nomogram for predicting incidence rate among patients with AP.

The following is an example of how the predictive model can be used: A male patient (67 points) with acute pancreatitis, no history of acute pancreatitis (48 points), and no stenosis or occlusion of the splenic vein (47 points) based on CT or MRI had the most significant site of swelling in the body-tail of the pancreas (72 points) and an MCTSI of 6 points (50 points), reaching a total score of 284 points. Based on the model, the probability of this patient to develop PSPH in the future is estimated at 23%. Conversely, for a male patient with pancreatitis and a previous history of AP who also exhibits splenic-vein stenosis or occlusion with the most obvious swelling of the body-tail, and an MCTSI of 10, the probability of developing PSPH in the future is very high (Figure 3).

## Verification of the model

**FIGURE 4:**
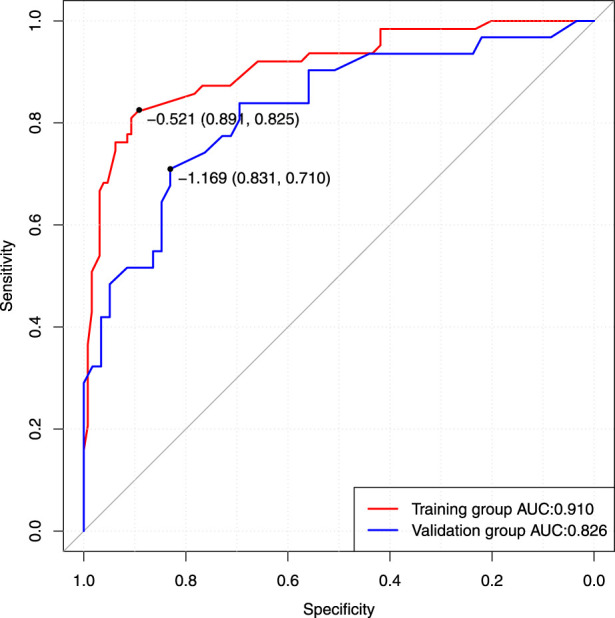
The receiver operating characteristic curve (ROC) was plotted based on the *p* values of the patients calculated using the screening model. Area under curve value was 0.910, with a sensitivity of 89.1% and a specificity of 82.5%. In the validation group, the C-index is 0.826. AUC, Area under curve.

**FIGURE 5:**
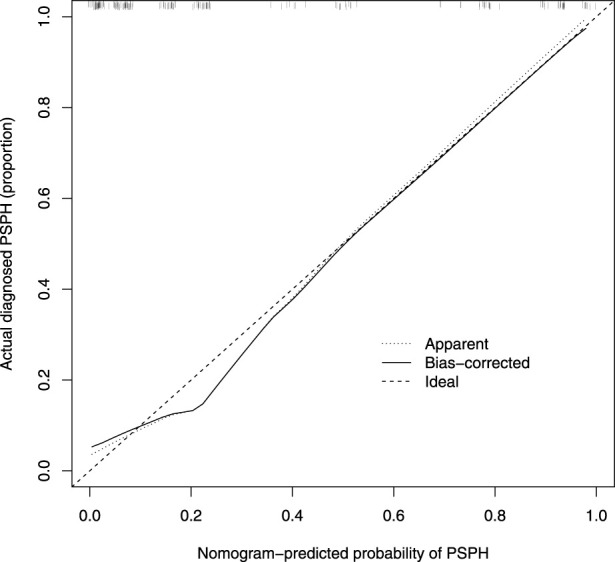
Calibration curve of the nomogram. The X-axis represented the predicted possible Pancreatic sinistral portal hypertension (PSPH) risk. The Y-axis represented the actual diagnosed PSPH. The diagonal dotted line meant a perfect prediction by an ideal model. The short-dashed line represented the apparent prediction of nomogram, and the solid line was bias-corrected by bootstrapping (B = 1,000 repetitions), indicating observed nomogram performance.

**FIGURE 6:**
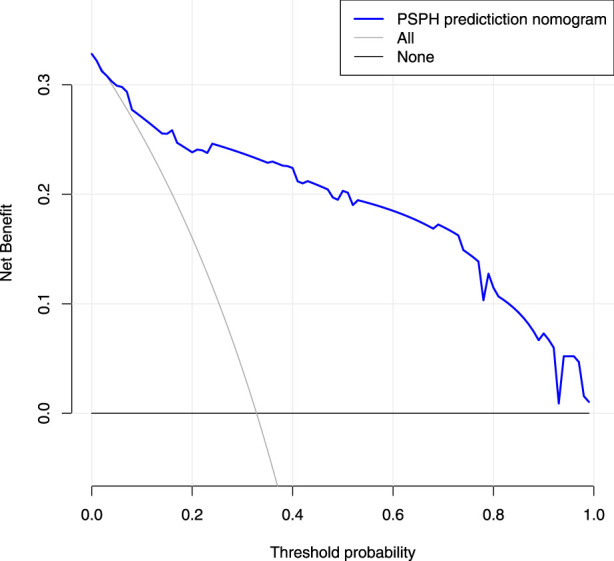
Decision curve analysis for the nomogram. The X-axis showed the threshold probability. The Y-axis measured the net benefit. The blue solid line represented the nomogram. The gray solid line represented the assumption that all subjects were Pancreatic sinistral portal hypertension.

Based on the results of cross verification, the predictive model showed good consistency, as evidenced by a c-index of 0.826, In the internal verification, the C-index was 0.898. Additionally, the calibration curve demonstrated good agreement between the predicted and actual PSPH values (Figures 4–6).

## Discussion

PSPH is a disorder that causes regional increases in portal vein pressure due to splenic-vein reflux. It is commonly associated with pancreatitis, and can lead to isolated gastric varices and splenomegaly. If left untreated, PSPH can lead to life-threatening upper gastrointestinal bleeding (Köklü et al., 2007; Xie et al., 2019; Ono et al., 2021). Preventing the onset of PSPH is crucial, as it is a curable form of portal hypertension. However, there are few studies that have assessed the risk factors for PSPH, and no predictive model of AP complicated with PSPH has been reported yet (Li et al., 2019). In this study, we identified male sex, recurrent AP, splenic-vein patency, swelling are located in the body-tail, and MCTSI as independent risk factors for PSPH onset. We used these findings to construct a nomogram that visualizes the results of the regression analysis. The model was internally verified and found to perform well, allowing for an accurate prediction of the probability of AP patients developing PSPH. Thus, the model developed in this study has significant implications for the prognosis of AP patients.

With the development of AP, various factors affecting the blood flow of the splenic vein appear, such as splenic-vein thrombosis, mechanical compression by pancreatic pseudocysts, and vascular-wall fibrosis resulting from repeated inflammatory stimulation. As a consequence of these factors, splenic-vein stenosis or occlusion may occur, leading to the occurrence of PSPH (Moossa and Gadd, 1985; Ito et al., 2008; Kul et al., 2018). It has been reported that the interval from the onset of AP to the onset of PSPH ranges from 10 days to 9 years. In the early stage, there may be splenic-vein stenosis or occlusion caused by inflammatory necrotic vascular-wall fibrosis, and in the late stage, thrombus and pseudocyst formations may mechanically oppress the splenic vein (Yu et al., 2022). We collected the AP history of each patient and conducted univariate analysis, which revealed a possible association between previous AP occurrences and PSPH (*p* < 0.001). Many studies have shown a strong correlation between the incidence of PSPH and male sex, and this may be related to the fact that men are more likely to develop splenic vein thrombosis (Roach et al., 2014). Furthermore, men often smoke (Shoar et al., 2018), which can cause atherosclerosis of the vascular-wall and is more likely to cause the formation of thrombus. Smoking has been found to increase the risk of vascular-wall atherosclerosis and thrombus formation (Flora and Nayak, 2019). In this univariate analysis, significant differences were observed between the male and female patients (*p* = 0.010), as well as between the patients with a history of smoking and those without (*p* = 0.021). These findings suggest that thrombus formation is one of the key factors contributing to the development of PSPH.

CT or MRI highly facilitates the diagnosis of pancreatic lesions (Kul et al., 2018; Xie et al., 2018; Xie et al., 2019). Complications of pancreatitis, such as necrotic accumulation, pseudocyst or thrombus formation, and patency of the splenic vein, depend on its evaluation. These factors are related to the occurrence of PSPH (Li et al., 2019). Currently, there is no established association between PSPH and the site of pancreatic swelling. However, our multivariate regression analysis revealed that the location of the lesion in the pancreatic body-tail (*p* < 0.001) is an independent factor linked to the occurrence of PSPH. It is considered that the splenic vein is tighter when the pancreatic body and tail are shaped, and it is easier to oppress the splenic vein when the pancreatic body and tail are swollen, and it is also related to the reflux of some small branches of the pancreatic vein located in the pancreatic body and tail into the splenic vein.

Yu et al. (2022) posited that the development of PSPH is independent of the etiology of AP. Conversely, Li et al. (2019) suggested a specific association between lipid-derived AP and PSPH, highlighting hypertriglyceridemia as a risk factor for moderate to severe AP. This study’s findings align with those of Chen, indicating that the emergence of PSPH is not directly linked to the etiology of AP. This led to the consideration that the incidence of PSPH might be more closely associated with the severity of pancreatitis. Of the seven studies that have investigated high serum triglyceride level as a potential factor affecting the severity of pancreatitis, five have found a possible association (Carr et al., 2016). The results of other studies have shown that BMI >25 kg/m^2^ increases the risk for critical AP (Dobszai et al., 2019; İnce et al., 2022). Although these indicators may increase the severity of AP, there was no significant relationship between PSPH and PSPH in this study. In addition, in a predictive model study based on thrombus and inflammatory markers, PT, D-dimer, CRP, and PCT were associated with the severity of pancreatitis (Han et al., 2022), only CRP was found to be significantly associated in our study. MCTSI scores have been widely used to assess the severity of AP (Alberti et al., 2021), and the PSPH group (*p* < 0.001) in our study scored higher among the data we included.

The identified independent risk factors for PSPH were utilized to develop a nomogram with high sensitivity and specificity. For individuals at elevated risk of PSPH, preemptive clinical intervention is recommended. Splenic vein thrombosis, a known complication of AP, has been associated with risk factors such as male gender, smoking, and elevated CRP levels (Toqué et al., 2015), findings that align with those of this study. Accordingly, clinicians should advise patients to cease smoking. In cases of splenic venous thrombosis, the use of anticoagulant therapy, previously viewed as having a low recurrence rate but high bleeding risk, remains contentious (De Stefano and Martinelli, 2010; Harris et al., 2013). Recent studies by Sissingh et al. (2023), however, advocate for the active use of low molecular weight heparin in treatment. The necessity of prophylactic low molecular weight heparin in AP patients without thrombosis formation has not been reported. In addition, we searched the time interval between the most recent AP episode and the diagnosis of PSPH. 16.7% of patients were diagnosed with PSPH at the time of hospitalization, 65.3% were diagnosed within 1 year after the most recent AP, and 18% were diagnosed more than 1 year after the most recent AP. In this study, more than 80% of PSPH patients can be diagnosed by abdominal CT or gastroscopy within 1 year. Therefore, for those who are at high risk of PSPH as indicated by the nomogram, we recommend that patients undergo semi-annual CT and gastrointestinal endoscopy, and pay attention to the situation of blood stool, avoid bleeding upper digestive tract massive bleeding, life-threatening.

There are also some limitations in this study. MCTSI is highly subjective, and different radiologists may assign different scores; hence, an internationally unified method is necessary for scoring the severity of pancreatitis. Moreover, the samples in this study were obtained from single centers, and data from multiple centers should be obtained in the future to verify the model externally.

In summary, this study identified male sex, splenic-vein stenosis or occlusion, recurrent AP, and swelling are located in the body-tail, and MCTSI is an independent risk factor for the occurrence of PSPH. The predictive model developed for AP complicated with PSPH may serve toward developing related preventive and therapeutic approaches.

## Data Availability

The raw data supporting the conclusion of this article will be made available by the authors, without undue reservation.
